# Effects of Long-Chain Omega-3 Polyunsaturated Fatty Acids on Endothelial Vasodilator Function and Cognition—Are They Interrelated?

**DOI:** 10.3390/nu9050487

**Published:** 2017-05-12

**Authors:** Julia C. Kuszewski, Rachel H. X. Wong, Peter R. C. Howe

**Affiliations:** 1 Clinical Nutrition Research Centre, School of Biomedical Sciences and Pharmacy, University of Newcastle, Callaghan, NSW 2308, Australia; Julia.Kuszewski@uon.edu.au (J.C.K.); peter.howe@newcastle.edu.au (P.R.C.H.); 2 Division of Research and Innovation, University of Southern Queensland, Raceview, QLD 4305, Australia

**Keywords:** long-chain omega-3 polyunsaturated fatty acids, endothelial vasodilator function, cerebral perfusion, cognitive function, cerebrovascular function, flow-mediated dilatation

## Abstract

Long-chain omega-3 polyunsaturated fatty acids (LCn-3 PUFA) may improve brain functions by acting on endothelial cells in the cerebrovasculature to facilitate vasodilatation and perfusion. The aim of this review is to explore this hypothesis by analyzing the effect of LCn-3 PUFA supplementation on systemic vasodilator and cognitive function and finding evidence to link LCn-3 PUFA intake, vasodilator function and cognition. Forty randomized controlled trials examining the effect of LCn-3 PUFA supplementation in humans on either endothelial vasodilator function or cognition were identified and pooled effects measured with a weighted analysis. Compared to placebo, LCn-3 PUFA tended to increase flow-mediated dilatation and significantly improved cognitive function. Emerging evidence links vasodilator dysfunction to cognitive impairment, but evidence that LCn-3 PUFA can improve cognition through enhancements of vasodilator function is still lacking. Further research is needed to determine: (1) whether LCn-3 PUFA can enhance dilatation of cerebral vessels; (2) if improvements in cerebrovascular responsiveness by LCn-3 PUFA are accompanied by cognitive benefits; and (3) the target population groups.

## 1. Introduction

Dementia is a progressive and irreversible impairment of cognitive function with a gradual onset that has profound consequences for both sufferers and their care providers, as well as being a great financial burden on society. With increasing life expectancy, dementia is a global public health problem with no cure or effective medical treatment currently available [1]. A growing body of evidence suggests that the initiation and progression of dementia is a consequence of poor cerebral vascular function and hypoperfusion [2,3,4,5,6]. The maintenance of cerebral blood flow (CBF) is crucial for normal brain functioning. A constant supply of oxygen and nutrients is vital, since the brain has a high metabolic need, but energy storage capacity is low [7]. CBF is augmented in response to local activities in brain regions to meet the metabolic needs of the neurons. This mechanism is termed neurovascular coupling (NVC) and is achieved by local vasodilatation of cerebral microvessels [8]. Impairments in NVC may lead to disturbances of substrate delivery to brain regions as well as removal of by-products of cerebral metabolism, thereby causing disruptions in the cerebral microenvironment [9]. Impaired CBF and NVC have been implicated in cognitive impairment and Alzheimer’s disease (AD) and no cure exists for this devastating disease [10]. Hence, there is a need for identifying lifestyle strategies to maintain adequate CBF so as to counteract accelerated cognitive decline in the aging population.

One such strategy is supplementation with LCn-3 PUFA, predominantly docosahexaenoic acid (DHA) and eicosapentaenoic acid (EPA), found in marine food sources such as fish, krill and algae [11]. Longitudinal observation studies have shown an inverse relation between fish intake and cognitive impairment [12]. Furthermore, there seems to be an association between the Omega-3 Index (EPA plus DHA as a percentage of total fatty acids in erythrocyte membranes), a recognized measure of an individual’s omega-3 status, and cognition [13,14]. Van der Wurff et al. demonstrated that typically developed Dutch adolescents with a higher Omega-3 Index have significantly higher information processing speed and lower impulsivity, a trait of executive function [14]. However, controlled intervention trials investigating the effect of LCn-3 PUFA supplementation on cognition show inconsistent results and the exact mechanisms by which LCn-3 PUFA aid cognitive function remain unclear [15].

In 2008, Sinn and Howe hypothesized that LCn-3 PUFA may affect cerebral functions through improvements in cerebral perfusion by acting on endothelial cells to facilitate vasodilatation [10]. A healthy endothelium is vital for the maintenance of blood circulation; it modulates fluidity, vascular tone and blood vessel integrity by producing and secreting numerous vasoactive compounds [10]. The most important of these is nitric oxide (NO), which is essential not only for vasodilatation, but also for maintaining blood vessel integrity by inhibiting cell adhesion, platelet aggregation and smooth muscle proliferation. Impairment of NO production or availability and the imbalance in the relative contribution of endothelium-derived relaxing and contracting factors are hallmarks of endothelial dysfunction, leading to impaired endothelium-dependent vasodilatation [16]. Restoring or improving endothelium-dependent vasodilatation might therefore be an important mechanism in order to improve cerebral function. Several studies have shown that LCn-3 PUFA supplementation can improve flow-mediated dilatation (FMD), a measure of systemic endothelial function [17,18]. However, it is unclear if improvements in systemic endothelial function translate to improvements in cerebral endothelial function that might lead to enhancements in cerebral function. Thus far, no study has validated the possible link among endothelial function, cerebral perfusion and cognition as hypothesized by Sinn and Howe and only a handful of studies have examined the effect of LCn-3 PUFA on cerebral vasodilator function in relation to cognitive function [19,20,21].

The aim of this review is to provide an update of Sinn and Howe’s hypothesis by: (1) presenting an overview of the current evidence for effects of LCn-3 PUFA supplementation on endothelial vasodilator function and cognition; (2) examining the relationship between vasodilator function and cognition; and (3) identifying gaps in literature and recommendations for future intervention studies.

## 2. Effects of LCn-3 PUFA Supplementation on Endothelial Vasodilator Function

LCn-3 PUFA have been shown to increase the synthesis of NO and prostacyclin in endothelial cells, with consequent vasodilator, anti-aggregatory and anti-inflammatory effects which help to maintain, restore or improve circulatory function [16,22]. Two meta-analyses published in 2012 assessed the effect of LCn-3 PUFA supplementation on endothelial vasodilator function. One reported that LCn-3 PUFA significantly increased FMD—a non-invasive index of vasodilator function in the systemic circulation—by 1.49% [17], while the other estimated a 2.30% increase in FMD [18]. We sought to capture any subsequent studies by searching and reviewing the scientific literature.

### 2.1. Literature Search Strategy and Study Selection

**Figure 1:**
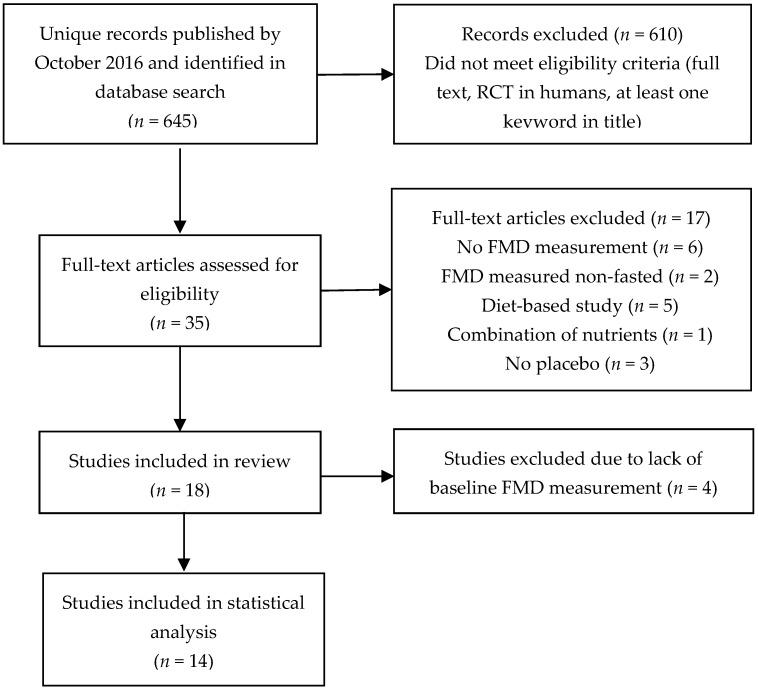
Flow chart of the study selection process for articles investigating the effect of long-chain omega-3 polyunsaturated fatty acid (LCn-3 PUFA) supplementation on endothelial vasodilator function.

PubMed and Scopus databases were searched for full text reports of randomized controlled trials (RCTs) in humans using the following key words: “long chain omega-3 fatty acids”, “fish oil”, “DHA” or “EPA” in combination with “endothelial function” and “flow-mediated dilatation (FMD)”. For consistency, FMD was limited to brachial artery ultrasound assessment. Exclusion criteria were: no measurement of FMD; measurement of FMD in a non-fasted state; not placebo-controlled; diet-based studies (no specified dose of EPA and DHA); and giving combinations of LCn-3 PUFA with other nutrients without using LCn-3 PUFA alone as a comparator. Six RCTs published between 2012 and October 2016 were identified in addition to 12 reported in the aforementioned meta-analyses (Figure 1).

### 2.2. Pooled Analysis

We performed statistical analyses (IBM SPSS Statistics 20, IBM Corp, Armonk, NY, USA) to see if LCn-3 PUFA supplementation has a significant beneficial effect on FMD (General Linear Model) and if this is influenced by the subject’s health status (healthy versus presence of chronic disease) or gender. Furthermore, using linear regression, we examined whether the relative change in FMD correlates with their baseline FMD, LCn-3 PUFA dose or study duration. The included studies were weighted according to the number of study participants.

### 2.3. Results of Statistical Analysis

In total, 18 cross-over or parallel design RCTs were included; they were conducted in a wide population range, including healthy adults, smokers or individuals suffering from chronic diseases such as type 2 diabetes, metabolic syndrome, hyperglyceridemia or systemic lupus erythematosus (SLE). Ten studies showed significantly improved endothelial vasodilator function (three within treatment group, and seven compared to placebo) after LCn-3 PUFA supplementation [23,24,25,26,27,28,29,30,31,32] (Table 1). For the statistical analysis, four studies were excluded because baseline FMD was not reported (analyzed: *n* = 14) [24,25,33,34]. Compared to placebo, absolute FMD increased by an average of 1.8 ± 2.4% (mean ± SD) after LCn-3 PUFA supplementation, representing a relative change in FMD of 33 ± 59% (*p* = 0.129, with baseline FMD as a covariate). This trend for FMD to increase after LCn-3 PUFA supplementation is consistent with the two previous meta-analyses reporting absolute increases of 1.5% and 2.3% respectively [17,18].

LCn-3 PUFA dose and study duration did not significantly correlate with the relative change in FMD. FMD at baseline, however, consistent with previous literature, was significantly inversely correlated with the relative change in FMD (*R* = −0.552, *p* = 0.001) [17,42,43]. Consistent with this finding, LCn-3 PUFA supplementation appears to be more beneficial for adults with chronic diseases compared to healthy adults without chronic diseases (trend: *p* = 0.081) as they are likely to have low FMD at baseline. Healthy individuals showed a relative increase in FMD of only 2.24%, while individuals with a chronic disease showed an increase of 33.6%. The improvement in FMD after LCn-3 PUFA supplementation is thus very limited for healthy individuals, possibly due to an already sound endothelial vasodilator function.

When we examined the treatment effect based on gender, studies with a >50% male population were more likely to show a beneficial effect of LCn-3 PUFA supplementation on FMD (*p* = 0.017). A study by Phang et al. demonstrated sex-specific effects of LCn-3 PUFA on platelet aggregation; men were more likely to benefit from EPA, while women were more responsive to DHA [44]. It might be that LCn-3 PUFA supplementation also exerts sex-specific effects on endothelial vasodilator function, causing more profound results in men, since participants were supplemented with a higher EPA than DHA dose (on average 1516 mg/day EPA vs. 983 mg/day DHA).

### 2.4. Possible Explanations for the Lack of Significant Results

Overall, LCn-3 PUFA supplementation improved absolute FMD by 1.8% compared to placebo, however this increase was not statistically significant, although a trend could be observed. In general, it is difficult to interpret absolute FMD values, since there is a lack of established normal FMD values across the lifespan and no universally agreed cut-off point between healthy and diseased individuals due to differences in data acquisition and data analyses between clinics [45]. However, a sustained increase in FMD may be of clinical importance not only for cardiovascular disease risk, but also for premature cognitive decline [46,47,48].

There are several study limitations that might, to some extent, explain the lack of significant results in some studies. For instance, 14 out of 18 studies did not measure the Omega-3 Index [23,25,26,27,28,29,30,31,32,33,35,36,38,39,49]. The index reflects the tissue EPA and DHA content in organs, thereby representing a person’s LCn-3 PUFA status. This is a very important measure, since individuals vary in their response to LCn-3 PUFA supplementation (age, gender, BMI) and the bioavailability of LCn-3 PUFA can also be affected by sodium, other supplements or drug consumption [13]. The index also influences the incorporation rate of EPA and DHA into erythrocyte membranes. People with lower Omega-3 Indices were shown to have higher incorporation rates [50]. In individuals with an already moderate baseline Omega-3 Index, further LCn-3 PUFA supplementation may only result in slight changes that are too small to reach significance. This might be one of the reasons why the studies by Sanders et al. [37] and Singhal et al. [40], which both measured a moderate Omega-3 Index (defined as an Omega-3 Index of 6–8% by Stark et al. [41]) in their participants, did not find any significant improvements in FMD after LCn-3 PUFA supplementation. Furthermore, most studies in this review (12 out of 18) did not account for the dietary LCn-3 PUFA intake at baseline and the eventual changes during the study, which might have also influenced the results.

There were a few studies with cross-over study designs. A limitation of these cross-over RCTs is that the wash-out period only comprised of 2–6 weeks, which, considering the slow turnover of LCn-3 PUFA in active stores, is insufficient. For instance, Cao et al. performed a study with 20 healthy participants who were supplemented with 2160 mg LCn-3 PUFA daily for 8 weeks. The study showed that four weeks after completion of the supplementation, the Omega-3 Index was still significantly higher than their baseline values [50], demonstrating the need for a long wash-out period of at least 10–12 weeks in cross-over studies.

Taken together, a trend towards improvement of FMD after LCn-3 PUFA supplementation can be observed. However, individual studies show inconsistent results, possibly due to differences between study populations (e.g., age, gender, health status, differences in baseline FMD and omega-3 status) and protocols (e.g., formulation, dose and duration of treatment). Considering the variation between studies, no further adjustment was made for differences in quality of design or performance of studies in the statistical analysis. Hence our observation can only be regarded as a tentative indication. Nevertheless, it serves as a guide to future research which could determine if EPA-rich supplementation benefits endothelial vasodilator function more in males than females and which population group (i.e., chronic disease risk) would benefit most from LCn-3 PUFA supplementation.

## 3. Effects of LCn-3 PUFA Supplementation on Cognitive Function

A meta-analysis by Mazereeuw et al. in 2012 assessed the effect of LCn-3 PUFA supplementation on cognition in older adults and concluded that it influences specific cognitive domains such as attention, processing speed and immediate recall [15]. We have also updated this meta-analysis.

### 3.1. Literature Search Strategy and Study Selection

**Figure 2:**
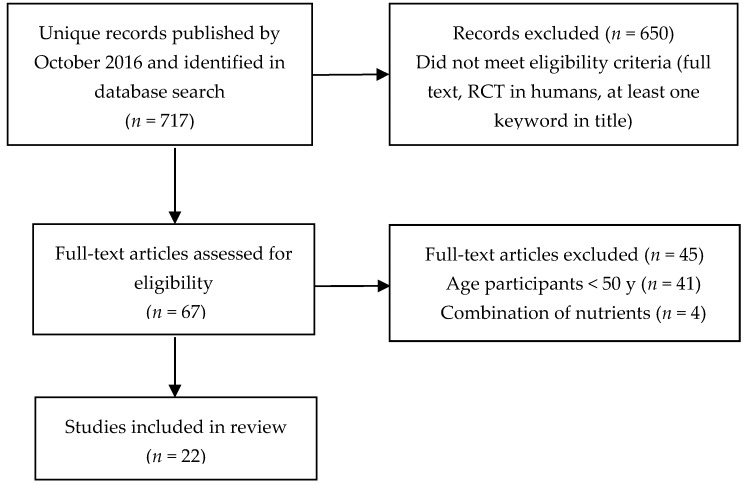
Flow chart of the study selection process for articles investigating the effect of LCn-3 PUFA supplementation on cognitive function.

PubMed and Scopus databases were searched for full text reports of RCTs performed in cognitively healthy or impaired adults above the age of 50 using the following key words: “long chain omega-3 fatty acids”, “fish oil”, “DHA” or “EPA” in combination with “cognition” and “cognitive function”. Exclusion criteria were: study participants <50 years old; not placebo-controlled; diet-based studies (no specified dose of EPA and DHA); and giving combinations of LCn-3 PUFA with other nutrients without using LCn-3 PUFA alone as a comparator. This review included 14 RCTs published between 2012 and October 2016 in addition to the eight RCTs reviewed by Mazereeuw et al. in 2012 (Figure 2).

### 3.2. Pooled Analysis

Similar to our FMD analysis, we used General Linear Modeling to see if LCn-3 PUFA supplementation significantly improved cognitive function, and, if so, whether it was influenced by the subject’s cognitive status (non-cognitively impaired, mild cognitive impairment (MCI) or AD) or gender. Furthermore, we examined whether there is a correlation between the outcome of the study as a dichotomous variable (improvement or no improvement) and EPA dose, DHA dose or study duration. The studies were weighted according to the number of study participants. However, for the reasons mentioned above, no further adjustment was made for differences in quality of design or performance of studies in the statistical analysis.

### 3.3. Results of Statistical Analysis

We included 22 RCTs that were conducted in either cognitively healthy (*n* = 12) or impaired older adults (age-related cognitive decline, MCI or AD, *n* = 10) (Table 2). Overall, the majority of studies (59%) observed an improvement in cognitive function after LCn-3 PUFA supplementation compared to placebo, which was statistically significant (*p* = 0.018) [21,51,52,53,54,55,56,57,58,59,60,61,62]. However, it is worth noting that these improvements were limited to certain cognitive domains such as information processing speed, executive function, recognition and recall memory.

The DHA dose was positively correlated with cognitive benefits (*R* = 0.300, *p* = 0.038). This finding is consistent with the study by Ismail in 2015 that compared the results of several LCn-3 PUFA intervention trials performed in participants with a cognitive health status ranging from non-cognitively impaired to mild AD [63]. He demonstrated that a minimum amount of DHA (approximately 800 mg/day) is required in order to observe significant improvements in cognitive function. Here, we did not find a significant correlation between EPA dose and cognitive benefits.

Furthermore, the length of the intervention trials adversely affected the cognitive benefits (*R* = −0.337, *p* = 0.022), which is consistent with the meta-analysis by Mazareeuw et al. [15]. One possibility might be confounding factors such as dietary changes during the intervention or treatment compliance. The longer is the study, the higher is the chance that participants will modify their diets. If participants in the placebo group start eating more fish during the trial than usual, their Omega-3 Index will rise and may confound the treatment comparison. Compliance is also an issue in longer intervention trials, with participants failing to consume their supplements regularly. Some long intervention studies included in this review did not report the compliance rate [49,64]. Others only counted the number of capsules to check for compliance, whereas the measurement of the Omega-3 Index would be far more specific, since it cannot be manipulated [62,65,66]. However, only five out of the 22 studies measured the Omega-3 Index [51,53,57,61,67]. Another issue with long term intervention trials, especially when including a large sample size, is that the inclusion criteria might not be as strict, leading to greater individual variability and confounding factors [64,65,66].

When we examined the treatment effect based on cognitive health, LCn-3 PUFA supplementation seemed to be most beneficial for individuals with MCI (*p* < 0.001). This finding is consistent with the meta-analysis by Mazareeuw et al. [15] as well as with the study by Freund-Levi et al. [49] that included adults with mild to moderate AD. Only the sub-group of participants with very mild cognitive dysfunction showed a significant reduction in Mini-Mental-State-Examination (MMSE) decline rate [49]. It seems to be crucial to start supplementation in older adults as soon as possible in order to improve cognitive function and counteract cognitive decline, since LCn-3 PUFA supplementation did not help individuals with AD.

Interestingly, in individuals without any cognitive impairment, most studies published before 2012 did not find any cognitive benefits, while most studies published from 2012 onwards did observe significant improvements in cognitive function. One reason for this might be the use of electroencephalography in these studies that measures the changes in event-related potentials in the brain, such as P300 latency, following supplementation. Examining the delay between cognitive stimuli presentation and the participants’ response time might have provided higher sensitivity and better precision to detect changes in cognitive function. For instance, the studies by Konagai et al. and Tokuda et al. supplemented healthy Japanese males with only 100 mg DHA per day and measured P300 latency during cognitive activity [21,56]. Even though the LCn-3 PUFA supplementation dose was very low, both studies demonstrated a significant decrease in P300 latency, which is a reflection of better information processing speed. Another example is the study by Kulzow et al. that used a DHA dose of 800 mg per day. They did not observe any improvement in composite memory scores, recall or recognition performance after LCn-3 PUFA supplementation. However, the recall of correct object-location-association was significantly higher in individuals supplemented with LCn-3 PUFA compared to controls [53].

When we examined the treatment effect based on gender, females appeared to benefit more from the effects of LCn-3 PUFA supplementation on cognitive function (trend: *p* = 0.071). This would be consistent with the study by Phang et al. that demonstrated sex-specific effects of LCn-3 PUFA on platelet aggregation. Women were shown to be more responsive to DHA, while men were more likely benefit from EPA [44]. It is possible that LCn-3 PUFA exerts similar sex-specific effects on cognitive function, since the participants were supplemented with more DHA than EPA (840 mg/day DHA vs. 618 mg/day EPA).

### 3.4. Study Limitations

Similar to the studies on endothelial vasodilator function, trials examining the effect of LCn-3 PUFA on cognitive function are missing the measurement of the baseline Omega-3 Index. Five out of 22 RCTs measured the Omega-3 Index [51,53,57,61,67], while nine out of 22 measured the percentage of EPA and DHA in total plasma fatty acid or phospholipids [19,21,49,56,59,60,68,69,70] (Table 2). Measuring the level of LCn-3 PUFA in plasma is less accurate as plasma fatty acids change in composition in response to a meal within hours, compared to the fatty acid composition in erythrocytes that takes weeks to change, making it suitable as long-term parameter [13].

Furthermore, only half of the studies (52%) recorded the dietary intake of LCn-3 PUFA at baseline and during the study.

In summary, LCn-3 PUFA supplementation significantly improved cognitive function; however, this improvement was restricted to certain cognitive domains. Moreover, females and individuals suffering from subjective memory complaints or MCI seem to benefit most. A sufficiently high DHA dose appears crucial to improve cognitive function. Future research should clarify if LCn-3 PUFA supplementation has only short-term effects or if the inverse correlation between intervention length and improvement in cognitive function is due to confounding factors.

## 4. Relationship between Endothelial Vasodilator Function and Cognition

Based on the reviewed literature, LCn-3 PUFA supplementation appears to benefit both endothelial vasodilator function and cognition, although the former requires further confirmation. Sinn and Howe hypothesized that LCn-3 PUFA supplementation can enhance cognitive function by acting on endothelial cells to influence vasodilatation, thereby boosting cerebral perfusion [10]. In this section of the review, we want to consider evidence for a relationship between vasodilator function and cognition and if this relationship might be partially mediated by effects on CBF.

### 4.1. Endothelial Dysfunction and Impaired Cognitive Function

Emerging evidence suggests that vascular dysfunction plays a role in the initiation and/or progression of dementia. For instance, Li et al. demonstrated that vascular risk factors (VRF) such as hypertension, diabetes, cerebrovascular diseases and hypercholesterolemia—which are associated with endothelial dysfunction—increase the risk of dementia incidence and that treatment of those VRFs can reduce this risk [71]. Furthermore, Vendemiale et al. has observed an association between an impaired FMD, indicating endothelial dysfunction, and MCI [47]. Another study by Dede et al. compared 24 AD patients with 25 age-matched controls and found that FMD was significantly lower in the AD patient group compared to controls [46]. Moreover, FMD was positively correlated with the MMSE score (*p* < 0.001). Furthermore, the study by Forman et al. showed a correlation between endothelial-dependent vascular responsiveness and neurocognitive performance among older patients with mild to severe CVD [72]. These studies strongly suggest a relationship between endothelial dysfunction and impaired cognitive function.

### 4.2. Decreased Cerebral Blood Flow and Impaired Cognitive Function

Apart from endothelial dysfunction, disturbances in CBF have also been associated with cognitive impairment. For instance, people suffering from MCI were shown to have a significantly decreased blood flow in the middle and anterior cerebral artery when compared to controls [6]. Moreover, the Rotterdam Study demonstrated that cerebral hypoperfusion precedes and possibly contributes to the onset of clinical dementia [4]. Alosco et al. demonstrated that a reduced CBF is associated with poorer memory and attention/executive function [73]. Impairments in CBF and NVC result in a diminished supply of nutrients and oxygen to the brain, which may lead to global neuronal dysfunction and/or neuronal death. This in turn may result in cognitive impairments and ultimately dementia [5].

### 4.3. Decreased Cerebral Blood Flow: A Possible Link between Endothelial Dysfunction and Impaired Cognitive Function?

**Figure 3:**
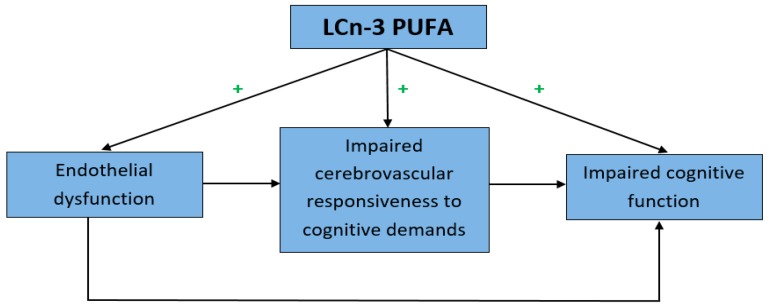
Schematic picture of the suggested relationship between endothelial dysfunction and impaired cognitive function, mediated by impaired cerebrovascular responsiveness to cognitive demands.

Systemic endothelial dysfunction and a reduced CBF have been associated with cognitive impairment. One possibility is that the relationship between endothelial dysfunction and cognitive impairment is mediated by effects on CBF. For instance, individuals with hypertension, who are likely to have endothelial dysfunction, were shown to have a reduced resting CBF as well as a lower CBF during cognitive tasks which was associated with poorer cognitive function [8,74]. Chen et al. suggested that the vascular endothelium is a critical pathway in functional NVC, since the interruption of endothelial signaling blocked the retrograde dilatation of pial arteries in response to a stimulus in rat brains, thereby limiting the capacity to increase CBF on demand [75]. The study by Lavi et al. also indicated a connection between systemic and cerebral endothelial function, since individuals with endothelial dysfunction were shown to have a disturbed CO2 vasoreactivity, which is a measure of the endothelium-dependent vasodilator responsiveness of cerebral vessels [76]. Therefore, endothelial dysfunction may result in impaired cerebral vasodilator responsiveness (CVR), leading ultimately to reduction of resting CBF and NVC. This in turn can cause impairments in cognitive function, since a constant blood supply with oxygen and nutrients is crucial for the maintenance of cellular integrity and information processing [10] (Figure 3).

In this review, we focus on the vasomotor function of the cerebral endothelium, which regulates the dynamic distribution of blood to meet regional cognitive demands. However, it is important to recognize that the endothelium in cerebral capillaries, representing the blood–brain barrier (BBB), is also crucial for brain function, as it regulates bilateral transport of substrates and waste products, thereby maintaining neuronal homeostasis [10]. If BBB integrity is impaired, pro-inflammatory and neurotoxic substances can leak into brain tissue, contributing to neuroinflammation and degeneration; increasing evidence links deficits in BBB integrity to onset of dementia [77,78]. LCn-3PUFA have been shown to ameliorate BBB damage induced by hypoxic/ischemic injury, preserve BBB ultrastructure and enhance tight junction protein expression in rats [79].

There is also increasing recognition that chronic systemic inflammation associated with obesity and metabolic syndrome plays an important role in endothelial dysfunction, contributing to impaired endothelial mediated vasodilatation [80]. Furthermore, there is increasing evidence linking chronic inflammation with cognitive decline [81]. The well documented anti-inflammatory actions of LCn-3 PUFA [82] could be contributing to improvements in cognitive performance by suppressing chronic inflammation and restoring endothelial vasodilator function in cerebral arterioles. Moreover, at capillary level, the anti-inflammatory properties of LCn-3 PUFA might counteract cognitive decline and reduce risk of dementia by maintaining BBB integrity and preventing leakage of neurotoxic substances that cause neuroinflammation. This important topic is addressed in more detail elsewhere [80,81,82].

## 5. Evidence to Link LCn-3 PUFA, Vasodilator Function and Cognition

### 5.1. Effects of Vasoactive Nutrients on Brain Function

Emerging evidence shows that certain vasoactive nutrients can improve systemic vasodilator function, enhance cerebrovascular function and also improve cognition. For instance, supplementing healthy older adults with an avenathramide-rich wild green oat extract for 24 weeks was found to improve systemic vasodilator function, measured by FMD, as well as CVR to hypercapnia, and marginally improved scores in the Stroop Color-Word Test [48]. Another vasoactive nutrient, resveratrol, has been shown to improve FMD in overweight, hypertensive adults [83]. Furthermore, resveratrol supplementation of post-menopausal for 14 weeks, elicited enhancements in CVR that correlated with improvements in cognitive function [84]. Consumption of nuts has also been shown to improve systemic endothelial vasodilator function after supplementation [85,86]. Barbour et al. recently demonstrated in cognitively healthy older adults that regular consumption of high-oleic peanuts (56 g/day for women, 87 g/day for men) improved CVR to hypercapnia and that this enhancement of cerebral endothelial function was accompanied by improvements in short-term memory, verbal fluency and processing speed [87].

### 5.2. Effects of LCn-3 PUFA Supplementation

LCn-3 PUFA supplementation may have similar effects as the other above-mentioned vasoactive nutrients. Based on the reviewed literature, LCn-3 PUFA supplementation can improve both endothelial vasodilator function and cognition. However, improvements appear limited to certain individuals. For endothelial vasodilator function, males and individuals with chronic diseases (with lower baseline FMD) appear to benefit most from LCn-3 PUFA supplementation. For cognitive function, females and individuals with MCI seem to benefit most. Furthermore, a sufficiently high LCn-3 PUFA intake is crucial to observe significant improvements. For cognitive function, an adequate DHA intake appears essential.

In none of the intervention trials reviewed above were the effects of LCn-3 PUFA supplementation on systematic endothelial vasodilator function and cognitive function measured simultaneously. Moreover, no study has determined whether: (1) LCn-3 PUFA supplementation has the same effects on the cerebral vasculature as in systemic vessels; and (2) there is a link between enhanced systemic vasodilator function, enhanced CBF and improvements in cognitive performance after LCn-3 PUFA supplementation. The effect of LCn-3 PUFA supplementation on vasodilator responsiveness may differ between systemic and cerebral vessels. Therefore, it is important to evaluate the effects of LCn-3 PUFA supplementation on cerebral vasodilator function as well. Only three RCTs have investigated effects of LCn-3 PUFA on both cerebrovascular function and cognitive function [19,20,21].

### 5.3. Effect of LCn-3 PUFA on Cerebrovascular Function

In 2012, Jackson et al. reported an effect of LCn-3 PUFA on CBF. This study was performed in 64 young university students (average age of 21 years) who were supplemented with 540 mg/day or 1080 mg/day LCn-3 PUFA (EPA: DHA ratio of 1:5) for three months [20]. Regional CBF was monitored in the pre-frontal cortex using near-infrared spectroscopy, which measures the concentrations of oxy- and deoxyhemoglobin, and was shown to be significantly increased during cognitive task performance. LCn-3 PUFA supplementation elicited a dose dependent enhancement of CBF; the dose of 1080 mg/day enhanced the CBF response during all cognitive tasks while enhancement by the 540 mg/day dose was limited to certain tasks. This is the first study to show that LCn-3 PUFA supplementation can improve NVC. However, the increased CBF was not accompanied by any improvements in cognitive performance. This might be due to the fact that the study participants were fairly young and at their cognitive peak, suggesting that only minor if any improvements in cognition were possible and too small to elicit a significant effect. The finding by Jackson et al. is consistent with the results of our analysis that only showed limited, non-significant improvements in non-cognitively impaired individuals (*p* = 0.303).

Konagai et al. conducted a study in healthy older adults to assess LCn-3 PUFA supplementation on cerebral perfusion and cognitive performance [21]. In this study, 42 healthy men aged 60–70 years were randomized to take krill oil (193 mg/day EPA and 92 mg/day DHA), sardine oil (491 mg/day EPA and 251 mg/day DHA) or a placebo for three months. The krill oil and sardine oil supplementation resulted in significantly greater changes in oxyhemoglobin concentration during a working memory task compared to placebo. Furthermore, in the krill oil group, significantly greater changes in oxyhemoglobin in the left frontal area during the calculation task were observed. Moreover, krill oil was shown to have beneficial effects on cognition by improving information processing speed (P300 latency). This study also shows an increase in CBF during cognitive tasks, representing an improved cerebral endothelial function and NVC. Furthermore, it is the first study to observe improvements in NCV being accompanied by improvements in cognitive function after LCn-3 PUFA supplementation in elderly individuals. In contrast, in a study of 86 elderly adults with subjective memory deficits published in 2016, Jackson et al. failed to show any significant effects of 1024 mg/day LCn-3 PUFA supplementation on cerebral perfusion or cognitive performance [19]. One possible explanation for this might be that the cognitive tasks used in the study were insufficiently sensitive to the neurophysiological changes resulting from LCn-3 PUFA supplementation.

The inconsistent results as well as the limited number of studies on the effect of LCn-3 PUFA supplementation on cerebral vasodilator function in combination with cognitive function make it difficult to draw a conclusion. Furthermore, each study uses different cognitive test batteries, making it difficult to compare results. Therefore, future studies might want to consider using standardized cognitive test batteries such as those offered by CANTAB or the NIH toolbox. Additionally, future studies should consider which population groups are most likely to benefit from the effects of LCn-3 PUFA supplementation on cerebrovascular function and cognition.

### 5.4. Gap in Literature

LCn-3 PUFA supplementation has been shown to improve systemic vasodilator function, NVC (CVR in response to cognitive stimuli) and cognition (Figure 3). However, these effects of LCn-3 PUFA were examined separately. Only one study so far has shown a link between enhanced cerebral vasodilator function and improvements in information processing speed in older adults without cognitive impairments [21]. More studies are needed to validate this link. Furthermore, there is no evidence that improvements in CVR due to LCn-3 PUFA supplementation result in an increased resting CBF. Reductions in resting CBF have been associated with poorer cognition and an increased risk of developing dementia [4,73]. The possible link between enhanced systemic and cerebral vasodilator function, CBF and cognition still needs to be established in a comprehensive battery of assessments (FMD, CBF at baseline, NVC (CVR to cognitive stimuli) and cognitive battery test) in a single study using a sufficiently high LCn-3 PUFA dose.

## 6. Future Perspective

### 6.1. Implications for Future Studies

Future studies establishing the link between vasodilator function, CBF and cognitive function after LCn-3 PUFA supplementation should take certain factors into account. Firstly, these studies should consider which population (e.g., chronic disease/gender/baseline Omega-3 Index) might benefit most from LCn-3 PUFA supplementation regarding endothelial vasodilator function, cerebral perfusion and cognition. For instance, hypertension and type 2 diabetes are associated with endothelial dysfunction and individuals suffering from those diseases have been shown to be predisposed to accelerated cognitive decline, making these individuals more likely to benefit from LCn-3 PUFA supplementation [88,89,90]. Moreover, LCn-3 PUFA appears to have sex-specific effects: the increase in FMD after LCn-3 PUFA supplementation was higher in men, while the cognitive benefits were more profound in women. Future studies should therefore consider the male: female ratio of their study population, depending on their study outcome.

Furthermore, it is important to measure the baseline omega-3 status of participants and studies should ideally include only those individuals with a baseline Omega-3 Index under a certain threshold (e.g., <6%, which is classified as low [41]), since these individuals may be more likely to benefit from the LCn-3 PUFA supplementation. Moreover, individuals with a certain amount of fish consumption (e.g., >1 serve of fish/week) should be excluded from the study. During the study, fish consumption should be monitored and limited in order to avoid any influence of dietary fish intake on the results of LCn-3 PUFA supplementation.

Secondly, the level of LCn-3 PUFA supplementation should be sufficient to elicit significant outcomes and should take account of potential differences between effects of EPA and DHA. Moreover, the ratio of LCn-6 PUFA to LCn-3 PUFA should also be taken into consideration. Observational studies showed that an increased omega-6/3 ratio is associated with cognitive decline (OR = 1.25–1.8) or dementia (OR = 1.1–1.6) [12]. Studies that used a high LCn-3 PUFA dose but did not find any significant results might have been confounded by a relatively high dietary omega-6 intake, resulting in insufficient changes in the omega-6/3 ratio after LCn-3 PUFA supplementation.

The last point to take into consideration in future studies is the intervention period. Whilst LCn-3 PUFA supplementation might influence endothelial vasodilator function fairly rapidly, a longer period may be necessary to detect changes in cognition. Future intervention studies are urgently needed to examine if the effects of LCn-3 PUFA supplementation on cognitive function are indeed only “short-term” (<6 months) or if there are other confounding factors leading to this result. Studies should have a long intervention period with regular follow-up to monitor the effects of LCn-3 PUFA supplementation over time. Dietary omega-3 and omega-6 intake should be closely monitored and participants with compliance below a certain threshold (e.g., <85%) should be excluded to rule out any confounding factors.

### 6.2. Testing the Hypothesis of Sinn and Howe

The hypothesis by Sinn and Howe that LCn-3 PUFA may aid cognition by acting on the endothelium to improve cerebrovascular function is still untested [10]. Therefore, our research group is currently conducting a 20-week randomized, double-blind, placebo-controlled dietary intervention trial to investigate the effect of 2 g/day LCn-3 PUFA supplementation (EPA: DHA ratio 1:4) on endothelial vasodilator function, cerebrovascular function and cognition in borderline hypertensive middle-aged to older adults (ACTRN12614000762651). Hypertensive adults are known to have endothelial dysfunction and are also predisposed to accelerated cognitive decline [8,88]. CBF velocity will be measured by transcranial Doppler (TCD) ultrasound, in the middle and posterior cerebral artery. The TCD device will also be used to measure CVR to hypercapnia, which assesses the endothelium-dependent vasodilator responsiveness of the cerebral vessels (cerebrovascular function). Furthermore, a neuropsychological test battery will assess cognitive performance during which CBF velocities are also continuously recorded to assess NVC. A comprehensive battery of assessments in a single study is beneficial for examining the link between vasodilator function, cerebrovascular function and cognition. The Omega-3 Index will be measured at baseline and at the end of the trial. The index will be used to monitor compliance as well as to examine if there is a correlation between the Omega-3 Index and outcome measures.

## 7. Conclusions

LCn-3 PUFA supplementation has been shown to have separate beneficial effects on systemic endothelial vasodilator function, CBF and cognitive function. In published studies, FMD was increased by an average of 1.8% (relative change of 32.7%) after LCn-3 PUFA supplementation compared to placebo and seems to benefit mostly men and individuals with chronic diseases (having impaired FMD). LCn-3 PUFA supplementation also resulted in significant cognitive benefits, although these appear restricted to certain cognitive domains. Women and individuals suffering from MCI seem to benefit most from LCn-3 PUFA supplementation. Literature shows evidence for a relationship between endothelial dysfunction and cognitive impairment which might be mediated by impairments in CBF. The hypothesis that LCn-3 PUFA supplementation may affect cerebral functions through improvements in cerebral perfusion by acting on endothelial cells to facilitate vasodilatation is still untested. Therefore, future intervention studies are urgently needed to ascertain whether: (1) beneficial effects of LCn-3 PUFA on systemic endothelial function are accompanied by improvements in cerebrovascular function; (2) improvements in cerebral vasodilator function due to LCn-3 PUFA are accompanied by improvements in cognitive performance; and (3) beneficial effects of LCn-3 PUFA supplementation on endothelial function and cognitive function can be sustained long term. High quality intervention studies with sufficiently high LCn-3 PUFA intakes are needed to clarify the length of the observed LCn-3 PUFA effects and which population group (males/females, cognitively impaired/unimpaired, healthy/suffering from chronic disease) would benefit most from LCn-3 PUFA supplementation.

## Figures and Tables

**Table 1:** Main characteristics of randomized placebo-controlled trials examining the effect of long-chain omega-3 polyunsaturated fatty acids (LCn-3 PUFA) supplementation on endothelial vasodilator function as measured by flow-mediated dilatation (FMD) (*n* = 18).

Reference	Year	Participants	Sample Size	Male/Female (%)	Intervention	Dose EPA/DHA (mg)	Duration (Weeks)	Baseline FMD (%)	Absolute Change in FMD * (%)	Outcomes	Baseline Omega-3 Index?
Cross-over study design											
Engler et al. (USA) [23]	2004	Children with FH or FCH, aged 9–19 years	20	NR	1200 mg/day DHA or placebo (corn oil)	0/1200	6 each, 6 weeks wash-out	6.3	1.1	Significantly increased FMD compared to placebo (*p* = 0.012)	No
Skulas-Ray et al. (USA) [34]	2010	Healthy adults with moderately elevated triglycerides, average age 44 years	26	88:12	850 mg/day or 3400 mg/day LCn-3 PUFA or placebo (corn oil)	465/375	8 each, 6 weeks wash-out	NR	NR	No significant difference in FMD between treatment and placebo group (*p* = 0.11)	Yes Low
						1860/1500					
Siasos et al. (Greece) [26]	2013	Healthy smokers, average age 28 years	20	65:35	1680 mg/day LCn-3 PUFA or placebo	920/760	12 each, 4 weeks wash-out	7.3	3.0	Significantly increased FMD compared to placebo (*p* < 0.05)	No
Tousoulis et al. (Greece) [27]	2014	Adults with metabolic syndrome, average age 44 years	29	52:48	1680 mg/day LCn-3 PUFA or placebo	920/760	12 each, 4 weeks wash-out	3.7	4.2	Significantly increased FMD compared to placebo (*p* < 0.001)	No
Zebrowska et al. (Poland) [28]	2015	Healthy endurance-trained male athletes, average age 23 years	13	100:0	1100 mg/day LCn-3 PUFA or placebo (lactose)	660/440	3 each, 2 weeks wash-out	10.3	5.2	Significantly increased FMD compared to placebo (*p* < 0.050)	No
Parallel study design											
Woodman et al. (Australia) [35]	2003	Adults with type 2 diabetes and hypertension, average age 61 years	30	76:24	4000 mg/day EPA or DHA or placebo (olive oil)	4000/0	6	3.1	−0.6	No significant difference in FMD between treatment (EPA and DHA) and placebo group	No
						0/4000					
Dyerberg et al. (Denmark) [33]	2004	Healthy males, average age 38 years	50	100:0	4000 mg/day LCn-3 PUFA or placebo	NR	8	NR	NR	No significant difference in FMD between treatment and placebo group	No
Hill et el. (Australia) [24]	2007	Overweight adults with ≥1 CVD risk factor, average age 52 years	35	39:63	1920 mg/day LCn-3 PUFA or placebo (sunflower oil)	360/1560	12	NR	NR	Significantly increased FMD compared to placebo (*p* < 0.010)	Yes Moderate
Shah et al. (USA) [25]	2007	Healthy adults, average age 32 years	26	65:35	500 mg/day LCn-3 PUFA or placebo (corn oil)	300/200	2	NR	NR	Significantly increased FMD within the treatment group (*p* = 0.036), no comparison to placebo	No
Wright et al. (UK) [32]	2008	Adults with SLE, average age 48 years	60	7:93	3000 mg/day LCn-3 PUFA or placebo (olive oil)	1800/1200	24	3.0	5.6	Significantly increased FMD within the treatment group (*p* < 0.001), no comparison to placebo; significant positive correlation between FMD and % DHA (*p* = 0.002) and % EPA (*p* = 0.026)	No Platelet FA very low
Rizza et al. (Italy) [31]	2009	Healthy adults (OPD), average age 30 years	50	50:50	1700 mg/day LCn-3 PUFA or placebo (olive oil)	1020/680	12	7.9	4.0	Significantly increased FMD compared to placebo (*p* < 0.010)	No
Wong et al. (China) [36]	2010	Adults with type 2 diabetes, average age 60 years	97	44:56	2680 mg/day LCn-3 PUFA or placebo (olive oil)	1680/1000	12	3.0	0.4	No significant difference in FMD between treatment and placebo group (*p* = 0.830)	No
Moertl et al. (Austria) [29]	2011	Adults with severe, nonischemic HF, average age 59 years	43	86:14	840 or 3360 mg/day LCn-3 PUFA or placebo (gelatine)	465/375	12	8.4	3.3	Within treatment group: 840 mg: trend to increase FMD (*p* = 0.070)	No
						1860/1500				3360 mg: increase in FMD (*p* = 0.010) No comparison with placebo	
Sanders et al. (UK) [37]	2011	Healthy adults, average age 55 years	312	39:61	450, 900 or 1800 mg/day LCn-3 PUFA or placebo (olive oil)	270/180	48	5.2	−0.6	No significant difference in FMD between treatment (all concentrations) and placebo group (*p* = 0.781)	Yes Moderate
						540/360					
						1080/720					
Hileman et al. (USA) [38]	2012	HIV infected males with moderate CVD risk, average age 51 years	35	100:0	1660 mg/day LCn-3 PUFA or placebo	930/730	24	3.2	−1.6	No significant difference in FMD between treatment and placebo group (*p* = 0.210)	No
Bello et al. (USA) [39]	2013	Adults with SLE, average age 47 years	85	9:81	3000 mg/day LCn-3 PUFA or placebo (corn starch)	1800/1200	12	12.5	−0.1	No significant difference in FMD between treatment and placebo group (*p* = 0.380)	No
Singhal et al. (UK) [40]	2013	Healthy adults, average age 28 years	274	40:60	1600 mg/day DHA or placebo (olive oil)	0/1600	16	8.4	−0.8	No significant difference in FMD between treatment and placebo group (*p* = 0.200)	Yes Moderate
Oh et al. (South Korea) [30]	2014	Healthy adults with hyperglyceridemia, average age 55 years	173	53:47	1000, 2000 or 4000 mg/day LCn-3 PUFA or placebo	Not specified	8	5.8	2.0	All LCn-3 PUFA concentrations significantly increased FMD compared to placebo (*p* < 0.050)	No

CVD = Cardiovascular disease, FA = fatty acids, FCH = familial combined hyperlipidemia, FH = familial hypercholesterolemia, HF = heart failure, NR = not reported, OPD = offspring of patients with type 2 diabetes, SLE = systemic lupus erythematosus, baseline omega-3 index was categorized as very low (≤4%), low (4–6%), moderate (6–8%) or adequate (>8%) based on the study by Stark et al. [41]. * Absolute change in FMD compared to placebo.

**Table 2:** Main characteristics of randomized placebo-controlled trials examining the effect of LCn-3.

Reference	Year	Participants	Sample Size	Male/Female (%)	Intervention	Dose EPA/DHA (mg)	Design and Duration	Outcomes	Baseline Omega-3 Index?
Non-cognitively impaired									
Johnson et al. (USA) [52]	2008	Healthy women, average age 68 years	24	0:100	800 mg/day DHA or placebo	0/800	Parallel-group, 4 months	Within treatment group: significantly improved verbal fluency (*p* = 0.030)	No
Van de Rest et al. (Netherlands) [68]	2008	Healthy adults, average age 70 years	300	55:45	400 or 1800 mg/day LCn-3 PUFA or placebo (sunflower oil)	226/176	Parallel-group, 6.5 months	No significant effect on any cognitive domains	No
						1093/847			Plasma FA: very low
Dangour et al. (UK) [66]	2010	Healthy adults, average age 75 years	744	55:45	700 mg/day LCn-3 PUFA or placebo (olive oil)	200/500	Parallel-group, 24 months	No significant effect on any cognitive domains	No
Nilsson et al. (Sweden) [54]	2012	Healthy adults, average age 63 years	38	30:70	2550 mg/day LCn-3 PUFA or placebo	1500/1050	CO, 5 weeks each with 5 week wash-out	Significantly improved word memory test performance at 60 min compared to placebo (*p* = 0.040)	No
Stough et al. (UK) [69]	2012	Healthy adults, aged 45–77 years	74	42:58	312 mg/day LCn-3 PUFA or placebo (soybean oil)	252/60	Parallel-group, 3 months	No significant effect on CDR cognitive outcomes measure compared to placebo	No Plasma PL: moderate
Konagai et al. (Japan) [21]	2013	Healthy men, average age 67 years	42	100:0	Krill oil, sardine oil or placebo (medium-chain triglycerides)	Krill oil: 193/92	Parallel-group, 3 months	Krill oil significantly decreased P300 latency compared to placebo (*p* = 0.030)	No Plasma FA in µg/mL
						Sardine oil: 491/251			
Witte et al. (Germany) [57]	2014	Healthy adults, average age 64 years	65	54:46	2200 mg/day LCn-3 PUFA + 15 mg vitamin E or placebo (sunflower oil)	1320/880	Parallel-group, 6.5 months	Significantly improved executive functions (26%, *p* = 0.005) compared to placebo, improvements in verbal fluency correlated with increase in EPA content (*p* = 0.009)	Yes Adequate
Chew et al. (USA) [65]	2015	Healthy adults, average age 73 years	2461	43:57	1000 mg/day LCn-3 PUFA + combination of vitamins or placebo	650/350	Parallel-group, 5 years	No significant effect on composite cognitive function score (*p* = 0.630)	No
Pase et al. (Australia) [67]	2015	Healthy adults, average age 59 years	70	46:54	960 mg/day LCn-3 PUFA or placebo (Sunola oil)	480/480	Parallel-group, 4 months	Increase in omega 3/6 ratio associated with improvement in spatial working memory response time (*p* < 0.050)	Yes Low
Tokuda et al. (Japan) [56]	2015	Healthy men, average age 60 years	69	100:0	400 mg/day LCn-3 PUFA or placebo (olive oil)	100/300	Parallel-group, 1 month	P300 latency significantly lower compared to placebo (*p* = 0.013)	No Plasma PL: adequate
Kulzow et al. (Germany) [53]	2016	Healthy adults, average age 62 years	42	52:48	2200 mg/day LCn-3 PUFA + 15 mg vitamin E or placebo (sunflower oil)	1320/880	Parallel-group, 6.5 months	Significantly improved recall of correct object-location-associations compared to placebo (*p* = 0.049)	Yes Adequate
Strike et al. (UK) [55]	2016	Healthy women, aged 60–84 years	27	0:100	1160 mg/day LCn-3 PUFA combined with multi-nutrients or placebo (oil blend)	160/1000	Parallel-group, 6 months	Within treatment group: significantly improved MOT latency (*p* = 0.038) and VRM immediate free recall (*p* = 0.029)	No
Cognitively impaired									
Freund-Levi et al. (Sweden) [49]	2006	Adults with mild to moderate AD, average age 73 years	174	48:52	2300 mg/day LCn-3 PUFA or placebo (corn oil)	600/1700	Parallel-group, 6 months	No significant effect on ADAS-Cog, MMSE or CDR scale Subgroup of very mild cognitive dysfunction: significant reduction in MMSE decline rate (*p* < 0.050)	No Plasma FA: moderate
Chiu et al. (Taiwan) [51]	2008	Adults with MCI or mild to moderate AD, average age 75 years	29	44:56	1800 mg/day LCn-3 PUFA or placebo (olive oil)	1080/720	Parallel-group, 6 months	Higher % EPA associated with better ADAS-cog scores (*p* = 0.003), Subgroup: adults with MCI treated with LCn-3 PUFA had an improved ADAs-cog score, compared to placebo (*p* = 0.030)	Yes Moderate
Quinn et al. (USA) [64]	2010	Adults with mild to moderate AD, average age 76 years	264	48:52	1000 mg/day DHA or placebo (corn oil)	0/1000	Parallel-group, 18 months	No significant effect on ADAS-cog, MMSE or CDR sum of boxes	No (only plasma DHA)
Yurko-Mauro et al. (USA) [58]	2010	Adults with age-related cognitive decline, average age 70 years	437	42:58	900 mg/day DHA or placebo (corn/soy oil)	0/900	Parallel-group, 6 months	Significant improvements in CANTAB PAL (*p* = 0.032), VRM immediate (*p* = 0.018) and delayed recall (*p* = 0.012) compared to placebo Cognitive changes significantly correlated with week 24 log plasma DHA levels	No (only plasma DHA)
Sinn et al. (Australia) [61]	2011	Adults with MCI, average age 74 years	40	67:33	EPA-rich or DHA-rich supplement or placebo (safflower oil)	1670/160	Parallel-group, 6 months	DHA significantly improved Initial Letter Fluency (*p* = 0.040)	Yes Low
						400/1550			
Lee et al. (Malaysia) [60]	2013	Adults with MCI, average age 65 years	36	25:75	1750 mg/day DHA rich fish oil or placebo (corn oil)	450/1300	Parallel-group, 12 months	Significant improvement in digit span, visual reproduction and delayed recall compared to placebo (*p* < 0.05 or *p* < 0.001)	No Plasma FA: very low
								Significant improvement in executive and attention function (*p* = 0.025) in intervention group	
								Significantly improved memory compared to placebo (*p* = 0.010)	
Eriksdotter et al. (Sweden) [59]	2015	Adults with AD, average age 74 years	165	48:52	2300 mg/day LCn-3 PUFA or placebo (corn oil)	600/1720	Parallel-group, 6 months	Significant positive association between plasma DHA levels and changes of total scores of ADAS-cog (*p* = 0.016)	No Plasma FA: moderate
Phillips et al. (UK) [70]	2015	Adults with CIND or AD, average age 71 years	76	45:55	1225 mg/day LCn-3 PUFA or placebo (olive oil)	600/625	Parallel-group, 4 months	No significant effect on cognitive function	No Plasma FA: adequate
Jackson et al. (UK) [19]	2016	Healthy adults with subjective memory deficits, aged 50–70 years	54	38:62	1024 mg/day DHA-rich fish oil or placebo (high oleic acid sunflower oil + 120 mg fish oil)	128/896	Parallel-group, 6 months	No significant effect cognitive function	No Plasma FA: low
Zhang et al. (China) [62]	2016	Adults with MCI, average age 75 years	219	35:65	2000 mg/day DHA or placebo (corn oil)	0/2000	Parallel-group, 12 months	Significant improvements in Full-Scale Intelligence Quotient (*p* = 0.039), Information (*p* < 0.001), Digit Span (*p* < 0.001) compared to placebo	No

AD = Alzheimer’s disease, ADAS-cog = Alzheimer’s disease assessment scale-cognitive subscale, CANTAB = Cambridge Neuropsychological Test Automated Battery, CDR = cognitive drug research, CIND = cognitive impairment no dementia, CO = cross-over, FA = EPA and DHA % in total plasma fatty acids, MCI = mild cognitive impairment, MMSE = mini-mental state examination, MOT = psychomotor response latency, P300 latency = rate of information processing, PAL = paired associate learning, PL = EPA and DHA % in plasma phospholipids, VRM = verbal recognition memory, baseline omega-3 index/total plasma fatty acids/plasma phospholipids was categorized as very low (≤4%/≤2.9%/≤3.8%), low (4–6%/2.9–4%/3.8–5.7%), moderate (6–8%, 4.0–5.2%/5.7–7.6%) or adequate (>8%/>5.2%/>7.6%) based on the study by Stark et al. [41].
